# Microbial degradation of isosaccharinic acid at high pH

**DOI:** 10.1038/ismej.2014.125

**Published:** 2014-07-25

**Authors:** Naji M Bassil, Nicholas Bryan, Jonathan R Lloyd

**Affiliations:** 1 Research Centre for Radwaste and Decommissioning and Williamson Research Centre for Molecular Environmental Science, School of Earth, Atmospheric and Environmental Sciences, University of Manchester, Manchester, UK; 2 National Council for Scientific Research–Lebanon (CNRS-L), Beirut, Lebanon; 3 National Nuclear Laboratory, Birchwood Park, Warrington WA3 6AE, UK

## Abstract

Intermediate-level radioactive waste (ILW), which dominates the radioactive waste inventory in the United Kingdom on a volumetric basis, is proposed to be disposed of via a multibarrier deep geological disposal facility (GDF). ILW is a heterogeneous wasteform that contains substantial amounts of cellulosic material encased in concrete. Upon resaturation of the facility with groundwater, alkali conditions will dominate and will lead to the chemical degradation of cellulose, producing a substantial amount of organic co-contaminants, particularly isosaccharinic acid (ISA). ISA can form soluble complexes with radionuclides, thereby mobilising them and posing a potential threat to the surrounding environment or ‘far field'. Alkaliphilic microorganisms sampled from a legacy lime working site, which is an analogue for an ILW-GDF, were able to degrade ISA and couple this degradation to the reduction of electron acceptors that will dominate as the GDF progresses from an aerobic ‘open phase' through nitrate- and Fe(III)-reducing conditions post closure. Furthermore, pyrosequencing analyses showed that bacterial diversity declined as the reduction potential of the electron acceptor decreased and that more specialised organisms dominated under anaerobic conditions. These results imply that the microbial attenuation of ISA and comparable organic complexants, initially present or formed *in situ*, may play a role in reducing the mobility of radionuclides from an ILW-GDF, facilitating the reduction of undue pessimism in the long-term performance assessment of such facilities.

## Introduction

High pH environments are present on Earth. They can be natural, for example, some geothermal springs and soda lakes, or man-made, for example, effluent ponds from paper pulping industries, lime working sites and closed structures made of cement. One such engineered structure is the deep geological disposal facility (GDF) that is being proposed for the safe disposal of radioactive waste. The largest volume of waste deposited into a GDF will be intermediate-level waste (ILW), which will be immobilised in steel containers backfilled with cementitious material to form a physical barrier in the form of a cement matrix (Nuclear Decommissioning Authority, 2014). The chemical and biological processes in and around this facility remain poorly understood, and therefore the impact of this facility on its surrounding environment remains uncertain. ILW contains substantial amounts of cellulosic material (Nuclear Decommissioning Authority, 2014), surrounded by hyperalkaline porewaters (Berner, 1992), and during disposal under these conditions, cellulose is known to be unstable (Van Loon and Glaus, 1997), and degrades chemically to short-chain organic acids (Glaus *et al.*, 1999), especially in the presence of radiation (Bouchard *et al.*, 2006). Although the nature and concentrations of the different products are influenced by temperature and the nature of the cellulose present (Haas *et al.*, 1967; Van Loon *et al.*, 1999), one of the main products of these degradation reactions is isosaccharinic acid (ISA) (IUPAC: 2,4,5-Trihydroxy-2-(hydroxymethyl)pentanoic acid) (Glaus *et al.*, 1999). At high pH, this molecule was found to sorb to cement in small amounts (Van Loon *et al.*, 1997), and is able to complex with a number of metals and radionuclides, particularly Ni(II) (Warwick *et al.*, 2003), Ca(II) (Vercammen *et al.*, 1999), Th(IV) (Vercammen *et al.*, 2001; Tits *et al.*, 2005), U(IV) (Warwick *et al.*, 2004), Eu(III) (Vercammen *et al.*, 2001; Tits *et al.*, 2005), Am(III) (Tits *et al.*, 2005) and Np(IV) (Rai *et al.*, 2003; Gaona *et al.*, 2008), making them potentially more mobile (Wieland *et al.*, 2002) and more likely to reach the biosphere.

The GDF has previously been considered to be an extreme environment where stresses including hyperalkalinity, radiation and radionuclide toxicity may play a role in limiting microbial colonisation. However, it is becoming clear that microbes may tolerate such extreme conditions (Chicote *et al.*, 2004; Rizoulis *et al.*, 2012); and as ILW will contain substantial amounts of organic molecules including cellulose and its alkali degradation products and other organic chelating agents like EDTA and nitrilotriacetic acid, used in remediation and decontamination processes, microbial colonisation should not be discounted. These organic molecules could be used as electron donors by microorganisms that respire a broad range of electron acceptors, including oxygen, nitrate, Fe(III) and sulphate. Given that natural alkaline environments harbour a wide diversity of microorganisms, it is safe to assume that the ‘evolved' GDF, resaturated with groundwater, might be a potential niche for a variety of specialist microorganisms including ISA-degrading organisms that can help reduce the transport of radionuclides from the GDF.

To date, only a few publications have addressed the microbial degradation of ISA, and these have focussed on aerobic conditions in paper pulping wastes. Strand *et al.* (1984) showed that *Ancylobacter aquaticus* is able to degrade ISA at pH values between 7.2 and 9.5 under aerobic conditions. Bailey (1986) identified two strains of aerobic bacteria that are able to degrade ISA at pH values between 5.1 and 7.2. On the other hand, Maset *et al.* (2006) have shown recently that biogeochemical redox progression from aerobic to Fe(III) reduction is possible when a sediment is incubated in the presence of ISA, although the degradation of ISA was not studied under these conditions. Therefore, the aim of this work is to study the microbial degradation of ISA under a number of biogeochemical conditions at high pH. This study will better inform GDF safety case assessments, and more specifically highlight the potential of microbial metabolism to decrease radionuclide mobility through the degradation of the complexant ISA.

## Materials and methods

### Sediment acquisition

Sediment samples were collected from a depth of ∼20 cm from the surface of a site, at Harpur Hill, Buxton, UK, that had been contaminated for decades by high pH legacy lime works. The sediments at the site generally have a pH between 11 and 12 and contain high calcium and silicate concentrations, analogous to a cementitious radioactive waste repository (Rizoulis *et al.*, 2012).

### Ca(α-ISA)_2_ preparation

Preparation of the Ca salt of α-ISA was performed following the procedure reported by Vercammen *et al.* (1999). Briefly, 500 ml of argon flushed double-distilled (dd) H_2_O was mixed with 50 g of α-lactose monohydrate and 13.6 g of Ca(OH)_2_ and left to react for 3 days under anaerobic conditions and at room temperature. The mixture was then boiled for 6 h, keeping the volume constant by adding dd H_2_O. The solution was then filtered while hot, and the volume of the filtrate reduced to ∼100 ml by boiling. The remaining solution was stored overnight at 4 °C. The white precipitate that formed was filtered and then washed sequentially with water, ethanol and acetone and dried overnight at 50 °C. The dry precipitate was redissolved at a ratio of 1.2 g in 100 ml of dd H_2_O by boiling. While still hot, the solution was filtered and the volume of the filtrate was reduced to ∼10 ml by boiling. The white precipitate that formed was washed sequentially with water, ethanol and acetone and then dried overnight at 50 °C.

### Aerobic cultures

Aerobic cultures were prepared in 100 ml serum bottles where 19.5 ml of minimal medium, containing 30 mM NaHCO_3_, 4.7 mM NH_4_Cl, 4.4 mM NaH_2_PO_4_.H_2_O, 1.4 mM KCl and 2 ml of mineral and vitamin solutions (Lovley *et al.*, 1984), was mixed with 200 μl of an inoculum of slightly turbid water overlaying the sediment samples from the Buxton site, and 2 mM Ca(ISA)_2_. The bottles were closed with a foam bung before autoclaving to allow the passage of air in and out of the bottles while preventing contamination from the surrounding environment. A ‘sterile' control along with a ‘no electron donor' control that did not contain any added carbon source were also prepared. All samples were prepared in triplicate and the pH was adjusted to 10 using 10 M NaOH before autoclaving. The cultures were left in a 20 °C incubator for the period of the experiment (until the stationary phase (assessed by turbidity) of the enrichment culture was reached). Samples (1 ml) were removed inside a laminar flow cabinet and the pH and absorbance at 600 nm were measured. The samples were frozen at −20 °C until the end of the experiment, when they were analysed by ion-exchange chromatography for ISA and a range of geochemical indicators. The test cultures were subcultured 4 times by taking 200 μl of the culture and adding it to freshly prepared medium under the same conditions. The data from the last subculture are shown.

### Nitrate-reducing cultures

Nitrate-reducing cultures were prepared following the same procedure as used with the aerobic cultures. However, 24 mM NaNO_3_, as the terminal electron acceptor (TEA), was added to the culture medium and the bottles were stoppered with rubber butyl stoppers and then degassed with N_2_ for 30 min before being autoclaved and the inoculum added. The same controls were used as in the aerobic culture experiments, in addition to a ‘no electron acceptor' control that contained 2 mM Ca(ISA)_2_ without NaNO_3_ added as the electron acceptor. Samples were taken with a 1 ml syringe and the pH and absorbance at 600 nm measured, and then they were frozen at −20 °C until they were analysed using ion-exchange chromatography. The test cultures were subcultured 3 times and the data from the last subculture are shown.

### Isolation of nitrate-reducing bacteria

The same minimal medium that was used for growth of the planktonic nitrate-reducing cultures was supplemented with 1% agar, pH adjusted to 10, flushed with N_2_ for 30 min, autoclaved and poured into petri dishes inside a laminar flow cabinet. After solidification of the agar, the plates were transferred into an anaerobic chamber and left upside down for 4 days. A subsample from the last subculture of the nitrate-reducing culture (the same one that was used for pyrosequencing studies) was spread onto the plates with a sterile disposable plastic spreader. The inoculated plate and a control plate were put in a sealed GasPak jar (Becton Dickinson, Franklin Lakes, NJ, USA), closed and incubated at 20 °C under anaerobic conditions. After 3 days, small transparent colonies were observed and five representative colonies were isolated using a sterile 5 μl microbiology loop and transferred to 20 ml of sterile minimal medium supplemented with 2 mM Ca(ISA)_2_ and 24 mM NaNO_3_. These bottles were incubated at 20 °C for 10 days, during which they were monitored for bacterial growth, pH change, ISA degradation and nitrate reduction. After the stationary growth phase was reached, 10 ml of the liquid culture was centrifuged at 4000 *g* for 10 min and the pellet was used for DNA extraction and microbial identification using Sanger sequencing of PCR amplified 16S rRNA gene.

### Fe(III)-reducing cultures

Fe(III)-reducing cultures were prepared and sampled in the same way as the nitrate-reducing cultures, except that 30 mmol l^−1^ of insoluble Fe(III) oxyhydroxide (ferrihydrite) (Lovley and Phillips, 1986) was added instead of NaNO_3_ as the TEA. The concentration of Fe(II) was determined spectrophotometrically after reaction with ferrozine (Lovley and Phillips, 1987). The test cultures were subcultured twice and data from the last subculture are shown.

### Sulphate-reducing cultures

Sulphate-reducing cultures were prepared and sampled following the same procedure as the nitrate-reducing culture, but the NaNO_3_ was replaced with 6 mM Na_2_SO_4_. These samples were not subcultured because, after 60 days of incubation of the primary enrichment culture, no reduction of sulphate or degradation of ISA was observed under the conditions imposed.

### Ion-exchange chromatography

The frozen samples were thawed at room temperature, vortexed for homogenisation and then centrifuged at 13 000 *g* for 5 min at room temperature to remove bacteria and any solid material. These samples were diluted 50 times and then analysed by ion-exchange high-performance liquid chromatography using a Dionex ICS5000 Dual Channel Ion Chromatograph with a conductivity detector (Thermo Fisher Scientific, Waltham, MA, USA). The samples were put in 2 ml glass vials with pre-split septa and cooled to 15 °C, and then 0.4 μl was injected into the chromatograph through a Dionex AS-AP autosampler. Molecule separation was achieved by passing the samples through a 250 × 0.4 mm Dionex AS11-HC capillary column with a 4 μm pore size, operating at 30 °C, with a typical operating pressure of 3400 psi and a flow rate of 0.015 ml min^−1^. The mobile phase used was a KOH solution, prepared in high-purity water, electronically injected to produce a gradient from 1 to 60 mM over a 38 min run time, followed by a 10 min re-equilibrium before the next injection. The chromatograph was calibrated at 4 points ranging from 0.5 to 30 mg l^−1^ for nitrate, nitrite and sulphate, 0.5–10 mg l^−1^ for acetate, formate, lactate, propionate and butyrate and 0.01–0.1 mM for ISA.

### 454 pyrosequencing

DNA was extracted from samples of aerobic, nitrate-reducing and Fe(III)-reducing cultures and the background sediment using the PowerSoil DNA isolation kit (MO BIO Laboratories, Carlsbad, CA, USA) according to the manufacturer's protocol. Pyrosequencing PCR was performed using the FastStart High Fidelity PCR System (Roche, Basel, Switzerland) and the 27F and 338R universal primers to cover the V1–V2 hypervariable regions of the 16S rRNA gene (Lane, 1991). The 338R primer with the sequence 5′-GCWGCCTCCCGTAGGAGT-3′ was used for all the samples. However, each sample had a unique F primer with a different barcode sequence to distinguish between the different samples. The F primers included the 454 Life Sciences (Bradford, CT, USA) adapter region with the sequence 5′-**CCATCTCATCCCTGCGTGTCTCCGACTCAG**-3′, followed by the 10 bp barcode sequence specific to each sample, where the background sediment had the sequence 5′-CTCGCGTGTC-3′, the aerobic sample 5′-CGTGTCTCTA-3′, the nitrate-reducing sample 5′-ATATCGCGAG-3′ and finally the Fe(III)-reducing sample had the sequence 5′-TCTCTATGCG-3′. The bold and underlines differentiate between the 454 Life Sciences adapter sequence and the barcode sequence in the forward primers we used for each sample. The barcode sequence was followed by the universal 27F primer sequence 5′-AGAGTTTGATCMTGGCTCAG-3′. The PCR mastermix contained, per sample, 40 μl sterile purified H_2_O, 5 μl of 10 × PCR reaction buffer, 1 μl dNTP mix, 0.8 μl of 25 μM 338R primer and 0.4 μl High Fidelity Enzyme Blend. To perform the PCR, 47.2 μl of this mastermix was transferred into a sterile 50 μl PCR reaction tube, where 0.8 μl of one of each barcoded 27F primer and 2 μl of DNA, taken from the sample corresponding to each specific barcode, were added to separate tubes. A negative and a positive control for the PCR reaction, which contained 2 μl sterile H_2_O or DNA extracted from *Geobacter sulfurreducens*, respectively, were also prepared. The PCR conditions were: initial denaturation at 95 °C for 2 min, followed by 35 cycles of denaturation at 95 °C for 30 s, primer annealing at 55 °C for 30 s and extension at 72 °C for 45 s, followed by a final extension step at 72 °C for 5 min. At the end of the PCR run, the whole PCR product was mixed with 12.5 μl of 5 × gel-loading dye, and 35 μl of the mixture was loaded on a 2% Tris-Acetate-EDTA/agarose gel. A 2000–100 bp ladder was also loaded on the gel that was run at 80 mV for ∼2 h. At the end of the run, the DNA bands were observed on a Gel Doc XR system (Bio-Rad Laboratories, Hercules, CA, USA) and the band corresponding to 400 bp size for each sample was excised from the gel. DNA extraction and cleanup from the excised gel were performed using a QIAquick Gel Extraction Kit (Quiagen, Limburg, The Netherlands), according to the manufacturer's protocol. DNA was quantified on a Nanodrop ND-1000 (Thermo Scientific) and all samples were diluted to 10 ng μl^−1^. The DNA product was then stored at 4 °C until it was sequenced using a 454 GS Junior pyrosequencing system (Roche), using the facility in the Faculty of Life Sciences, University of Manchester.

### Pyrosequencing data analysis

Analysis of the raw 454 pyrosequencing data was done using the Quantitative Insights Into Microbial Ecology pipeline (Caporaso *et al.*, 2010b). Sequences were first assigned to the different samples by using the barcode sequences provided, and sequences outside the 300–400 bp range were removed along with the reverse primer sequence, using the split_library.py script. Chimeric sequences were identified using the usearch 6.1 programme (Edgar *et al.*, 2011) and the identify_chimeric_seqs.py script. Chimeric sequences were filtered out of the data using the filter_fasta.py script. Operational taxonomic units (OTUs) were picked from these sequences and compared at 97% similarity with the May 2013 release of greengenes OTU reference using the usearch 6.1 programme (Edgar, 2010) through the pick_otus.py script. The most abundant OTU sequence was chosen as a representative sequence, using the pick_rep_set.py script, and assigned to taxonomy based on the greengenes reference database (McDonald *et al.*, 2012) using the Ribosomal Database Project Naive Bayes classifier v 2.2 (Wang *et al.*, 2007), with the confidence level set at 80% through the assign_taxonomy.py script. The sequences were then aligned to the greengenes core reference alignment (DeSantis *et al.*, 2006) using PyNAST (Caporaso *et al.*, 2010a) through the align_seqs.py script. Aligned sequences were then filtered using the filter_alignement.py script, and a phylogenetic tree was built through the make_phylogeny.py (Price *et al.*, 2009). An OTU table was built through the make_otu_table.py and convert_biom.py scripts and was used to calculate and plot the α-diversity (based on the number of OTUs) and the % 16S rRNA gene reads in each sample using the OriginPro v 9 software (OriginLab, Northampton, MA, USA) .

### Sanger sequencing of 16S rRNA genes from pure cultures

DNA was extracted from the pellets of the five isolates of the nitrate-reducing culture using the PowerSoil DNA isolation kit (MO BIO Laboratories) according to the manufacturer's protocol. The 16S rRNA gene was amplified by PCR using the TaKaRa Ex Taq Polymerase (EMD Millipore, Billerica, MA, USA) and universal primers 8F (with the sequence 5′-AGAGTTTGATCCTGGCTCAG-3′) and 1492R (with the sequence 5′-TACGGYTACCTTGTTACGACTT-3′) for the whole 16S rRNA gene (Turner *et al.*, 1999). The PCR mastermix contained, per sample, 36.7 μl sterile purified H_2_O, 5 μl of 10 × Ex buffer, 4 μl dNTP mix, 1 μl of 25 μM 8F and 1492R primers and 0.3 μl Ex TaKaRa Taq polymerase. To perform the PCR, 48 μl of this mastermix was transferred into a sterile 50 μl PCR reaction tube and 2 μl of the extracted DNA was added. A negative and a positive control for the PCR reaction, which contained 2 μl sterile H_2_O or DNA extracted from *Geobacter sulfurreducens*, respectively, were also prepared. The PCR conditions were: initial denaturation at 94 °C for 4 min, followed by 30 cycles of denaturation at 94 °C for 30 s, primer annealing at 55 °C for 30 s and extension at 72 °C for 1.5 min, followed by a final extension step at 72 °C for 5 min. The purity of the amplified product was determined by running 8 μl of the PCR product with 2 μl of 5 × gel loading dye on a 1% Tris-Acetate-EDTA/agarose gel against a 2000 bp DNA ladder. The PCR product (∼1500 bp) was cleaned from the PCR salts using the ExoSap (Affimetrix, Santa Clara, CA, USA) protocol, where 0.08 μl Exonuclease I (20 U μl^−1^; New England Biolabs, Ipswich, MA, USA), 1.5 μl rAPid Alkaline Phosphatase (1 U μl^−1^; Roche) and 1.42 μl dd H_2_O were mixed with 3 μl of the PCR product, incubated at 37 °C for 30 min and then at 80 °C for 15 min. Nucleotide sequences were determined by the dideoxynucleotide method by cycle sequencing using an ABI Prism 377 DNA sequencer (Applied Biosystems, Carlsbad, CA, USA). A presequencing PCR step was performed using the ABI Prism BigDye Terminator Cycle Sequencing Kit v 3.1 (Applied Biosystems) and the 16S gene universal 8F primer. The PCR mastermix contained 0.75 μl Terminator BigDye, 3.65 μl 5 × buffer, 0.15 μl 8F primer and 14.95 μl dd H_2_O. The DNA template from the cleanup step was quantified on a Nanodrop ND-1000 (Thermo Scientific) and diluted to get 40 ng of DNA per PCR reaction. The PCR conditions were: initial denaturation at 96 °C for 2 min, followed by 30 cycles of denaturation at 96 °C for 40 s, primer annealing at 55 °C for 15 s and 60 °C for 3 min, followed by a final extension step at 72 °C for 5 min. The PCR product was then precipitated following the ethanol/EDTA precipitation protocol supplied with the kit. The data produced were analysed against the NCBI database, using the Basic Local Alignment Search Tool (megaBLAST) programme package and matched to known 16S rRNA gene sequences.

## Results

**Figure 1:**
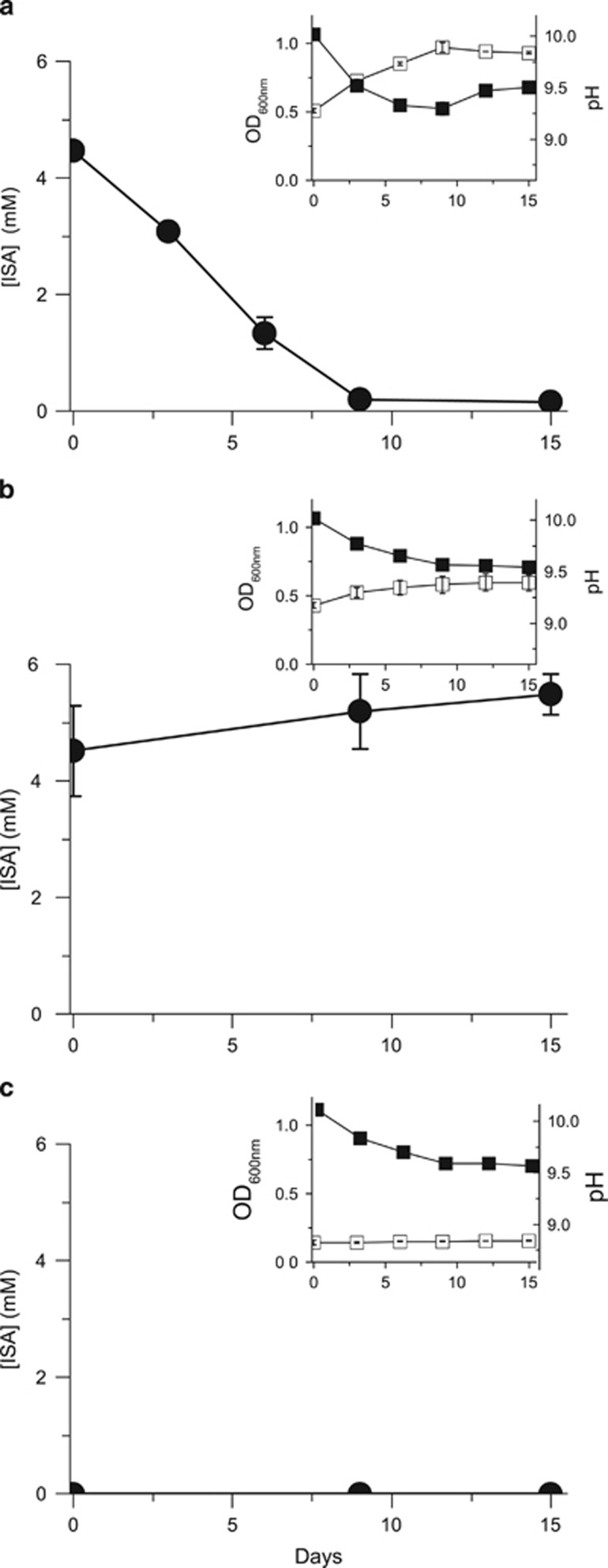
ISA biodegradation by aerobic microbial cultures at a starting pH of 10. (**a**) Test sample containing active microbial cells, (**b**) sterile (autoclaved) control and (**c**) a control containing an active inoculum but no added ISA as the sole carbon source and electron donor. Upper panels show bacterial growth (OD_600 nm_) (□) and pH (▪). The lower panels show the concentration of ISA (●) in mM.

**Figure 2:**
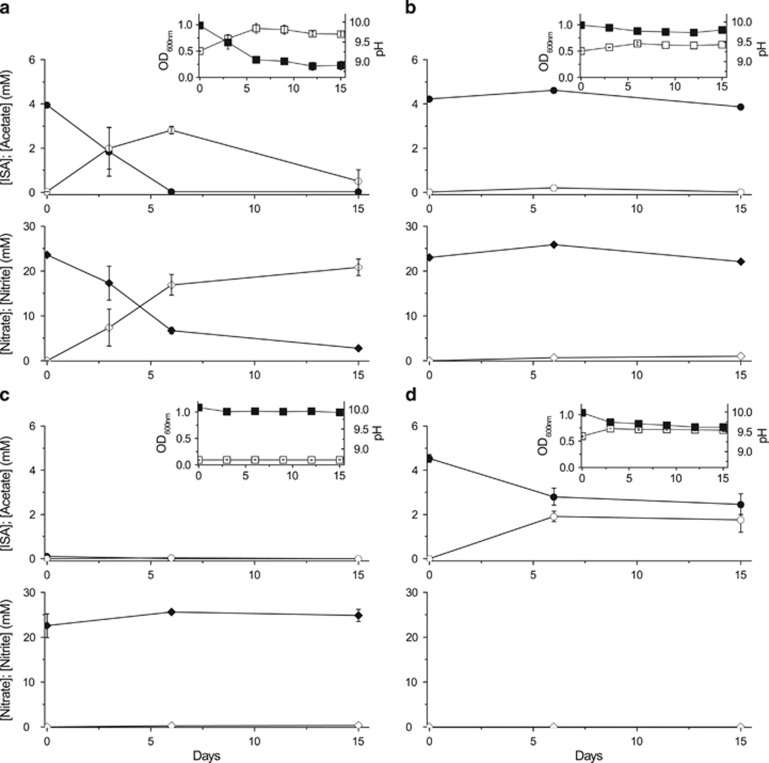
ISA biodegradation by nitrate-reducing microbial cultures at a starting pH of 10. (**a**) Test sample containing active microbial cells, (**b**) sterile (autoclaved) control, (**c**) a control containing an active inoculum but no added ISA as the sole carbon source and electron donor and (**d**) a control containing an active inoculum but no added nitrate as the electron acceptor. Upper panels show bacterial growth (OD_600 nm_) (□) and pH (●). The middle panels show concentrations of ISA (●) and acetate (○) in mM. The lower panels show concentrations of nitrate (♦) and nitrite (⋄) in mM.

**Figure 3:**
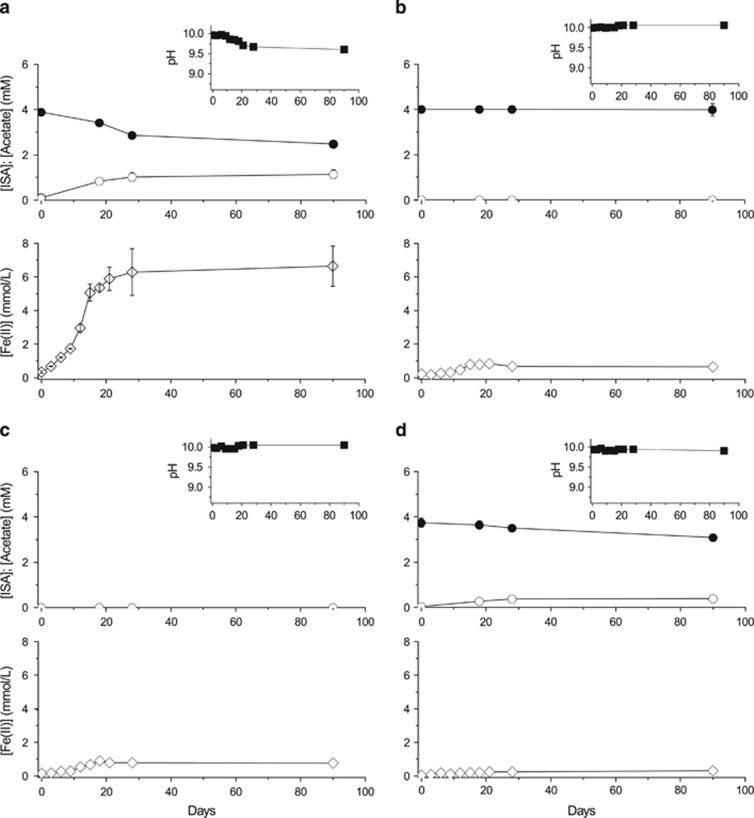
ISA biodegradation by Fe(III)-reducing microbial cultures at a starting pH of 10. (**a**) Test sample containing active microbial cells, (**b**) sterile (autoclaved) control, (**c**) a control containing an active inoculum but no added ISA as the sole carbon source and electron donor and (**d**) a control containing an active inoculum but no added Fe(III) as the electron acceptor. Upper panels show pH (●) change with time. The middle panels show concentrations of ISA (●) and acetate (○) in mM. The lower panels show concentrations of Fe(II) (⋄) in mmol l^−1^.

To determine whether alkaliphilic or alkalitolerant microbial communities, capable of ISA degradation, are present in a high pH analogue site for concrete-based ILW, samples from the Harpur Hill (Buxton, Derbyshire, UK) historic lime workings were inoculated into defined minimal medium containing ISA as the sole electron donor and either oxygen (as air), nitrate, Fe(III) or sulphate as an electron acceptor. Under aerobic conditions (Figure 1), all the added ISA was degraded within 9 days of incubation. This ISA degradation was accompanied by an increase in turbidity and a drop in pH indicating bacterial growth and the production of CO_2_ as a product of ISA degradation (the latter linked to acidification of the medium via the formation of bicarbonate). Under nitrate-reducing conditions (Figure 2), a broadly similar rate of degradation of ISA was recorded; however, complete removal of the substrate was noted within only 6 days of incubation that was coupled to the reduction of ∼71% of the added nitrate to nitrite. Concomitant with the ISA degradation, substantial amounts of acetate (∼3 mM) were produced between the initial time point and day 6 of incubation. The produced acetate was then degraded between days 6 and 15 of incubation, and part of the remaining nitrate was reduced to nitrite. Similar to the aerobic samples, the turbidity increased under nitrate-reducing conditions whereas the pH dropped, confirming bacterial growth and metabolism. Furthermore, bacteria in the nitrate-reducing cultures were also able to grow and cause a drop in the pH of the medium at a range of temperatures (10, 20 and 30 °C) in cultures incubated at starting pH values of 10 and 11 (Supplementary Figure S1), whereas no growth was observed at a starting pH of 12 even after 27 days of incubation. Under Fe(III)-reducing conditions (Figure 3), only ∼36% of the added ISA was degraded, even after 90 days of incubation. This was accompanied by the reduction of ∼21% of the added Fe(III) to Fe(II) and the production of acetate as a breakdown product. As expected, the pH dropped only slightly because of the small amount of ISA that was degraded by microbial activity. The sulphate-reducing cultures (Supplementary Figure S2) did not show any signs of bacterial growth, ISA degradation or sulphate reduction even after 60 days of incubation under the same conditions as used in the previous cultures. These results are consistent with previous data, generated using similar high pH microcosms prepared from similar sediments from the Buxton field site, that did not support sulphate reduction when lactate and acetate were supplied as electron donors, even after 20 weeks of incubation (Rizoulis *et al.*, 2012). Therefore, these enrichment cultures showed a sequential degradation of ISA, where the rate and extent of ISA biodegradation generally decreased as the reduction potential of the TEA decreased (in the order aerobic≈nitrate>Fe(III), with no reduction of sulphate detected at pH 10).

**Figure 4:**
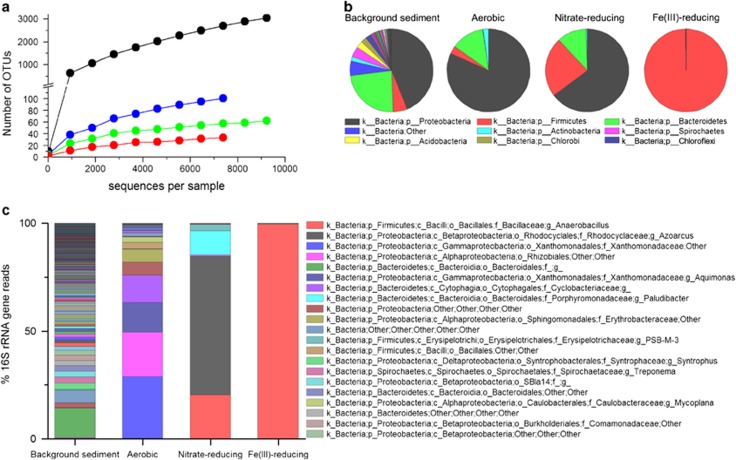
Microbial diversity from 454 pyrosequencing analyses of the starting inoculum, aerobic, nitrate- and Fe(III)-reducing ISA degrading cultures. (**a**) The α-diversity plot showing the number of OTUs in the background sediment (black), aerobic cultures (blue), nitrate-reducing cultures (green) and the Fe(III)-reducing cultures (red) with respect to the number of reads. (**b**) Pie charts showing the bacterial phyla present in each sample. (**c**) Bar chart showing the bacterial genera in the different samples that were tested. All taxa that show <2% expression are shown in the graph, but not indicated in the legend.

PCR-based 16S rRNA gene analyses of the microbial communities in these enrichment cultures using 454 pyrosequencing showed that the sediment used for the starting inoculum contained a very complex bacterial community that became far less diverse under the highly selective growth conditions employed (Figure 4). This was evident in the α-diversity plot (Figure 4a) that showed a complex bacterial community in the background sediment (with 3060 OTUs) that declined sequentially as the enrichment cultures progressed through a series of biogeochemical processes in the order aerobic (101 OTUs)>nitrate-reducing (64 OTUs)>Fe(III)-reducing conditions (33 OTUs). At the phylum level (Figure 4b), the background sediment and the aerobic cultures were dominated by Gram-negative bacteria, in particular Proteobacteria (44% and 81% of the 16S rRNA gene reads detected, respectively) and Bacteroidetes (24% and 13%, respectively), with a very low number of Gram-positive Firmicutes detected (6% and 3%, respectively). However, under anaerobic conditions, there was a dramatic shift in the dominant community structure, with the nitrate-reducing cultures containing an increased proportion (24%) of Gram-positive Firmicutes, and these also dominated the Fe(III)-reducing cultures (comprising almost 100% of the bacterial community). At the genus level (Figure 4c), the high microbial diversity in the background sediment and the aerobic cultures masked dominance of any particular bacterial genus in these samples; furthermore, very few OTUs were identified down to the genus level; however, these samples contained common soil bacteria such as *Aquimonas*. The bacterial genera that dominated the nitrate-reducing cultures were the facultative anaerobic denitrifiers *Azoarcus* (65%), and the obligate anaerobes *Paludibacter* (12%) and *Anaerobacillus* (21%), with the latter almost completely dominating the Fe(III)-reducing enrichment cultures (99.5%). Further analysis was carried out using the megaBLAST programme package that matched the pyrosequencing data to known 16S rRNA gene sequences to find the closest known relatives to the OTUs identified in the nitrate- and Fe(III)-reducing enrichment cultures. This analysis showed that the OTU that dominated the nitrate-reducing cultures (65%) was a close relative (at 97% identity) of the facultative denitrifying bacterium *Aromatoleum aromaticum* (which belongs to the *Azoarcus* cluster) (Kuhner *et al.*, 2005), and the close relative *Azoarcus buckelii* (Mechichi *et al.*, 2002). The second most dominant OTU in the nitrate-reducing cultures (21%), which dominated the Fe(III)-reducing cultures with almost 100% coverage, was a close relative (at 94% identity) to the bacteria *Anaerobacillus macyae*(previously known as *Bacillus macyae*) (Santini *et al.*, 2004) and *Anaerobacillus alkalidiazotrophicus*(previously known as *Bacillus alkalidiazotrophicus*) (Sorokin *et al.*, 2008). The last OTU in this sample was most closely related to *Paludibacter propionicigenes* (Ueki *et al.*, 2006) (at 96% identity), a strictly anaerobic propionate and acetate producer.

Single colonies were isolated from agar plates prepared under nitrate-reducing conditions and all five isolates were able to degrade ISA and reduce nitrate at a similar rate to the mixed cultures under nitrate-reducing conditions (data not shown). Furthermore, analysis of the partial 16S rRNA gene sequence (1050 bp) of the isolates showed that all the isolates showed 98% similarity to the bacterium *A. alkalidiazotrophicus* and the related bacterium *A. macyae*. The former is an alkaliphilic strictly fermentative bacterium with no respiratory chain that can fix dinitrogen (Sorokin *et al.*, 2008), whereas the latter is an arsenate and nitrate respiring bacterium that is known to utilise acetate as an electron donor (Santini *et al.*, 2004). Interestingly, these bacteria were not the most dominant in the nitrate-reducing samples based on 454 pyrosequencing results; however, they dominated in the Fe(III)-reducing enrichments.

## Discussion

This study shows for the first time that bacteria are able to degrade ISA (as a sole carbon and energy source in minimal media) at high pH (∼10) under a wide range of biogeochemical conditions, and can couple ISA metabolism to the bioreduction of a number of TEAs relevant to an evolving ILW-GDF. Furthermore, the rates of ISA biodegradation, TEA reduction and the bacterial diversity of the cultures were proportional to the reduction potential of the TEA, in the order aerobic≈nitrate>Fe(III), with no reduction of sulphate detected at pH 10. Collectively, these results are consistent with the observation that a range of anaerobic processes can support ISA oxidation, and that decreasing the reduction potential of the respiratory process redox couple results in progressively more streamlined microbial communities dominated by more specialist microorganisms, as noted previously in high pH environments (Burke *et al.,* 2012). In this case, we noted the enrichment of nitrate- or Fe(III)-reducing Gram-positive Firmicutes that may have a competitive advantage under strongly reducing conditions at high pH.

During the operational phase of the GDF, a dry oxygen-rich environment will dominate in the cementitious wasteform and a substantial amount of cellulosic material will be deposited in the ILW. After closure, the GDF will be resaturated with groundwater and anoxic conditions will develop. The pH of the GDF is expected to start at around 13.3 because of the dominance of the cementitious chemistry (Berner, 1992). Under these high pH conditions, substantial amounts of ISA will be formed from the alkali hydrolysis of the deposited cellulose (Askarieh *et al.*, 2000). Although the pH inside the GDF is expected to start at much higher values than those that we tested, these starting hyperalkaline pH values will drop to ∼10 over prolonged periods of time (Berner, 1992), well within the range of pH values shown to support ISA degradation in our study. Furthermore, at shorter times, even when the pH is still very high inside the GDF, there will be a pH gradient surrounding it that will result in regions of decreasing pH from hyperalkaline to ambient with increasing distance from the GDF. Therefore, any ISA produced within the ILW would pass through regions of pH similar to those tested in this study as it migrates to the geosphere.

Very few previous studies have dealt with the biological degradation of ISA, and they have exclusively targeted ISA biodegradation under aerobic conditions (Strand *et al.,* 1984; Bailey, 1986). In this respect, and to our knowledge, this is the first study that deals with the bacterial degradation of ISA at high pH and under anaerobic conditions when maximal rates of ISA formation are expected to occur because of the wasteforms becoming saturated with hyperalkaline groundwater post closure. Furthermore, ISA degradation in the presence of nitrate, which can be present in some wasteforms in the GDF, took place at a similar rate to that recorded in aerobic systems (Figures 1 and 2). Furthermore, Fe(III), which can form in the GDF by corrosion of iron associated with the wasteforms or iron-containing engineering structures like rock bolts and steel canisters, also supported ISA oxidation by bacteria (Figure 3). The lack of evidence for bacterial degradation of ISA under sulphate-reducing conditions does not negate the ability of sulphate-reducing bacteria to degrade ISA and couple this degradation to the reduction of sulphate. It is possible that such processes could take place very slowly over longer periods of time within the GDF, or in the surrounding geosphere (after diffusion of the ISA) where a lower pH groundwater dominates. Here, the ΔG for the sulphate reduction reaction will be lower than at pH 10 (Rizoulis *et al.*, 2012) and active sulphate-reducing bacteria may be present. This is important as many deep groundwaters in the United Kingdom and other countries considering GDF options can contain appreciable concentrations of sulphate.

It is probable that microbial metabolism of ISA will become more pronounced as the pH drops with time and distance from the GDF, and as the extant geosphere microbial communities adapt to the high pH conditions in and around the GDF over prolonged periods of time. Furthermore, in these scenarios potential ISA degraders could be sourced from the deep geosphere, or could survive in the initial hyperalkaline pH as recalcitrant spores, and then germinate when the conditions are more suitable for growth. It should be noted that Gram-positive Bacilli, well known to sporulate as a survival mechanism (Madigan *et al.*, 2010), dominated many of our experiments. As ISA-metabolising bacteria have the potential to decrease radionuclide mobility, via the degradation of soluble radionuclide-ISA complexes, such microbial processes should be included in performance assessments focussing on ILW-GDFs. This work, and follow-on studies, can help address significant gaps in our current understanding of the long-term evolution of these unusual engineered subsurface environments. Of particular interest could be investigations of microbial ISA degradation under more realistic subsurface conditions *in situ* (for example, using underground laboratory facilities) and potentially using cellulose-bearing ILW under prolonged incubation. Collectively, these studies can play an important role in removing unnecessary pessimism in performance assessment evaluations for GDFs.
