# Supplementary figures and images for: Microbial degradation of isosaccharinic acid at high pH
Source: ISME J. 2014 Jul 25;9(2):310–20. doi: 10.1038/ismej.2014.125 (PMC4303625; doi:10.1038/ismej.2014.125)

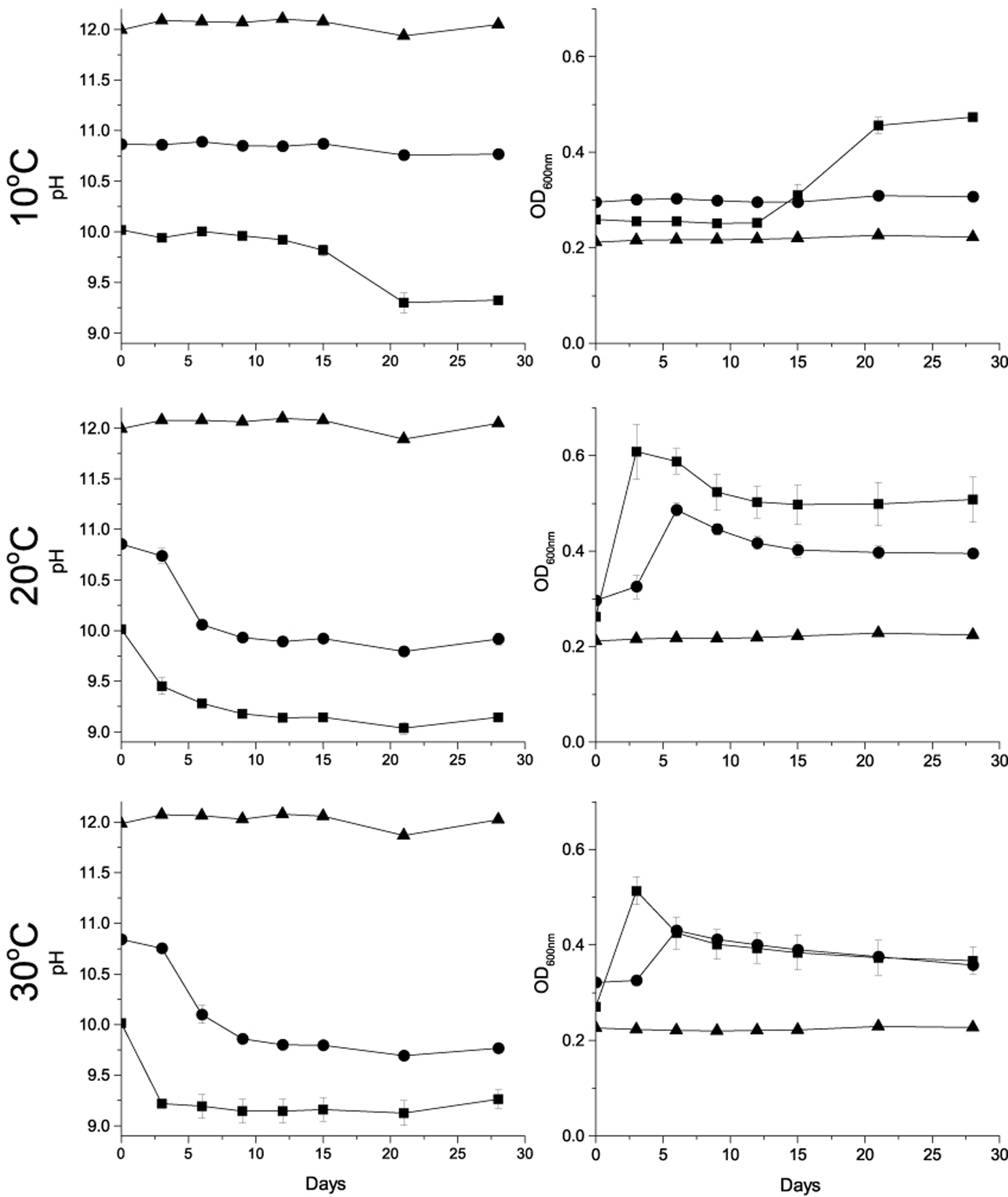

Supplement: Supplementary Figure S1 [file ismej2014125x1.tif]

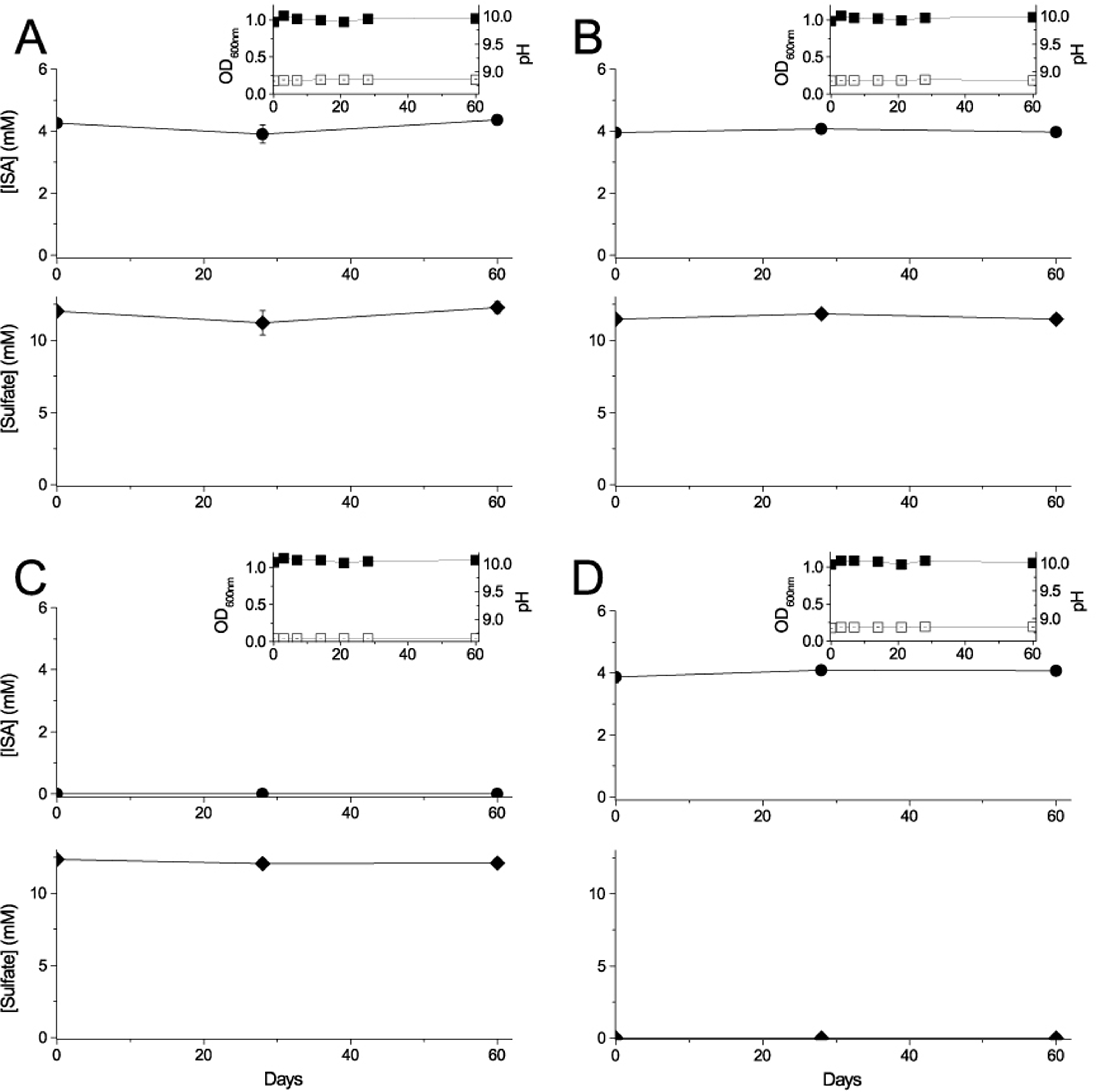

Supplement: Supplementary Figure S2 [file ismej2014125x2.tif]
