# Supplementary material for: Microbial degradation of isosaccharinic acid at high pH
Source: ISME J. 2014 Jul 25;9(2):310–20. doi: 10.1038/ismej.2014.125 (PMC4303625; doi:10.1038/ismej.2014.125)
Supplement: Supplementary Figure Legends [file ismej2014125x3.doc]

Supplementary Figure Legends

Supplementary figure S1: Nitrate-reducing culture bacteria grown at pH 10, 11 and 12 at different temperatures 10, 20 and 30^o^C. (A) represents pH and absorbance change over time at 10^o^C. (B) represents pH and absorbance change over time at 20^o^C. (C) represents pH and absorbance change over time at 30^o^C. **■** represents the samples prepared at a starting pH of 10, ● represents the samples prepared at a starting pH of 11, and **▲** represents the samples prepared at a starting pH of 12.

Supplementary Figure S2: ISA biodegradation by sulfate-reducing microbial cultures at a starting pH of 10. (A) Test sample containing active microbial cells, (B) sterile (autoclaved) control, (C) a control containing an active inoculum but no added ISA as the sole carbon source and electron donor and (D) a control containing an active inoculum but no added sulfate as the electron acceptor. Upper panels show bacterial growth (OD_600nm_) (□) and pH (**■**) change with time. The middle panels show concentration of ISA (●) in mM. The lower panels show the concentration of sulfate (◆) in mM.
